# Light simulation and yield assessment of lettuce cultivated under agrivoltaic systems in Northern Thailand

**DOI:** 10.1038/s41598-025-23083-y

**Published:** 2025-11-13

**Authors:** Suwimon Wicharuck, Wahyu Nurkholis Hadi Syahputra, Nuttapon Khongdee, Tasanee Pripanakul, Ar Man, Napassawan Wongmongkol, Nilubon Luangchosiri, Yupa Chromkaew, Nanchaphorn Udomsri, Marcus Nagle, Chatchawan Chaichana

**Affiliations:** 1 Office of Research Administration, Chiang Mai University, 50200 Chiang Mai, Thailand; 2 Energy Technology for Environment Research Center, Faculty of Engineering, Chiang Mai University, 50200 Chiang Mai, Thailand; 3 Department of Mechanical Engineering, Faculty of Engineering, Chiang Mai University, 50200 Chiang Mai, Thailand; 4 Agrotechnology Study Program, Faculty of Agriculture, University of Jember, 68121 Jember, Indonesia; 5 Department of Highland Agriculture and Natural Resources, Faculty of Agriculture, Chiang Mai University, 50200 Chiang Mai, Thailand; 6 Graduate Master’s Degree Program in Energy Engineering, Department of Mechanical Engineering, Faculty of Engineering, Chiang Mai University, 50200 Chiang Mai, Thailand; 7 Department of Plant and Soil Sciences, Faculty of Agriculture, Chiang Mai University, 50200 Chiang Mai, Thailand; 8 Agricultural Research & Development Program, Central State University, 45384 Wilberforce, OH USA

**Keywords:** Agrivoltaic system, Greenhouse, Light availability, Lettuce production, Light simulation, Ecology, Ecology, Plant sciences

## Abstract

This study aimed to (i) simulate and validate light intensity under the Agrivoltaic system (AVS) and (ii) evaluate the feasibility of lettuce (*Lactuca sativa* L.; varieties: ‘Butter Head’, ‘Green Oak’ and ‘Red Oak’) plantation under AVS. . Two planting systems located in Northern Thailand were compared: (i) “Greenhouse (GH)” = lettuce planting inside the greenhouse conditions (control plot), and (ii) “Agrivoltaic system (AVS)” = lettuce planting underneath a 10 kW photovoltaic installation. Sunlight estimation was performed using Rhinoceros 3D software with Grasshopper Plugin and Ladybug Tools, and the model accuracy was validated. For the experimental study, lettuce growth (plant height and canopy width) and yields (fresh and dry weight) were collected every 7 days after transplanting. The fresh weight (FW) was directly measured after harvest. The samples were oven-dried at 65 °C and the dry weight (DW) was recorded. The results showed high accuracy of the model to predict sunlight under specific conditions with R^2^ of 0.82. Plant height and canopy width under the AVS were not significantly different from those in the GH system. Significant higher FW and DW were observed for the GH system (FW = 61.6 g plant^−1^ and DW = 2.9 g plant^−1^) as compared to those in the AVS (FW = 42.2 g plant^−1^ and DW = 2.2 g plant^−1^). These results demonstrate that the Rhinoceros 3D software has the potential to predict sunlight and that sunlight under AVS is adequate for growing crops.

## Introduction

Solar farming systems have become more popular around the world. These systems typically require large areas of land for installing photovoltaic (PV) systems. For example, a 1-MW solar farm needs approximately 2.4 ha of land^1^. Routine operations, including system monitoring, cleaning, and vegetation control, are required to ensure the optimal performance and longevity of the PV systems. These include specific costs for the different operations, such as the cost of weed management underneath the PVs, which is documented at around 663 USD ha^−1^ in the USA^2^. Subsequently, innovative solutions are necessary to reduce maintenance costs and make better use of the land under solar panels. One approach is AgriVoltaic Systems (AVS), which combine energy production with agricultural practices underneath the panels^3^. AVS can optimize both land use efficiency and resource utilization, especially in regions with limited arable land. However, growing crops under AVS requires consideration of several key factors, including light availability, water management, and crop selection.

Sunlight provides the energy that plants need for photosynthesis, the process by which CO_2_ and H_2_O are converted into chemical energy essential for crop growth and biomass accumulation^4–8^. Three light characteristics are important for plant growth: light quality, intensity and duration. Light quality refers to sunlight in the spectral wavelengths between 400 and 700 nm, known as photosynthetically active radiation (PAR)^9^. Light intensity is the amount of photons that plants receive within this PAR range per unit area and time, measured as photosynthetic photon flux density (PPFD, µmol m^−2^ s^−1^). In addition, photoperiod (or light duration) represents the length of daily light (number of hours) in a day. The cumulative amount of light within the PAR range over a given period is defined as daily light integral (DLI, mol m^−2^d^−1^)^10,11^.

Each crop requires different ranges of DLI; for example, lettuce (*Lactuca sativa* L.) needs recommended DLI values of 12–17 mol m^−2^ d^−1 12^ and optimal DLI values of strawberries (*Fragaria × ananassa*) is 20–25 mol m^−2^ d^−1 13^. Shading from PV panels can reduce availability of sunlight due to panel height and spacing. These low light conditions can lead to plant irregularity symptoms such as cell elongation and increased leaf area, known as the shade avoidance syndrome^14^. Some crops can adjust to low levels of light intensity by increasing their rate of vegetative growth^15^, whereas an excess of sunlight (over the plant threshold) may damage crop quality and yield^16^.

Cossu et al.^17^ pointed out that DLI under different PV cover ratios (PV_R_) were lower than outside conditions, with values of 34.8, 24.4, 18.6, 13.2, 12.0 and 7.1 mol m^−2^ d^−1^ under outside, conventional greenhouse, 25% PV_R_, 50% PV_R_, 60% PV_R_ and 100% PV_R_, respectively. Many studies have been conducted to investigate the potential of ground-mounted PVs for crop cultivation, such as lettuce, tomatoes tomatoes (*Solanum lycopersycum* L), potatoes (*Solanum* *tuberosum*), winter wheat (*Triticum* *aestivum*) and soybeans (*Glycine max* L.)^18–21^. López-Díaz et al. (2020) found that shading from solar panels at levels of 0, 15, 30, and 50% resulted in a decrease in tomato yield from 18.8 to 11.5 kg m^−2^, in a comparison between AVS and open field conditions. Lee et al. (2022) reported lower yields of soybean and rice under AVS, while potato yield was higher under AVS than in open field condition.

Lettuce is one edible vegetable plants that tolerates reduced light environments in terms of morphological adaptation^22,23^, making it a promising applicant for AVS conditions. Accurate prediction of light distribution is essential for optimizing AVS performance. Computer software, such as Rhinoceros 3D, can be a useful tool for sunlight simulation under different conditions. According to several studies ^24,25^, the software can be used to predict sunlight availability at different levels of vertical farming shelves. The results indicated the possibility of using the software to predict sunlight intensities. Forecasting sunlight under AVS helps to understand and design an appropriate orientation of plants to be suitable for specific light conditions in the year.

Although AVS has been studied extensively in temperate regions, limited data are available for tropical countries where high solar irradiance and seasonal variations can be useful for the performance of the system. Few studies have combined validated light modeling with year-round prediction of crop yields and resource use analysis in tropical AVS contexts. Therefore, this study aimed to (i) simulate and validate light intensity under the AVS and (ii) evaluate the feasibility of lettuce plantation under AVS in Thailand. The results will help to understand the potential of AVS for sustainable food production, especially lettuce, in tropical environments.

## Materials and methods

### Experimental setup

**Fig. 1:**
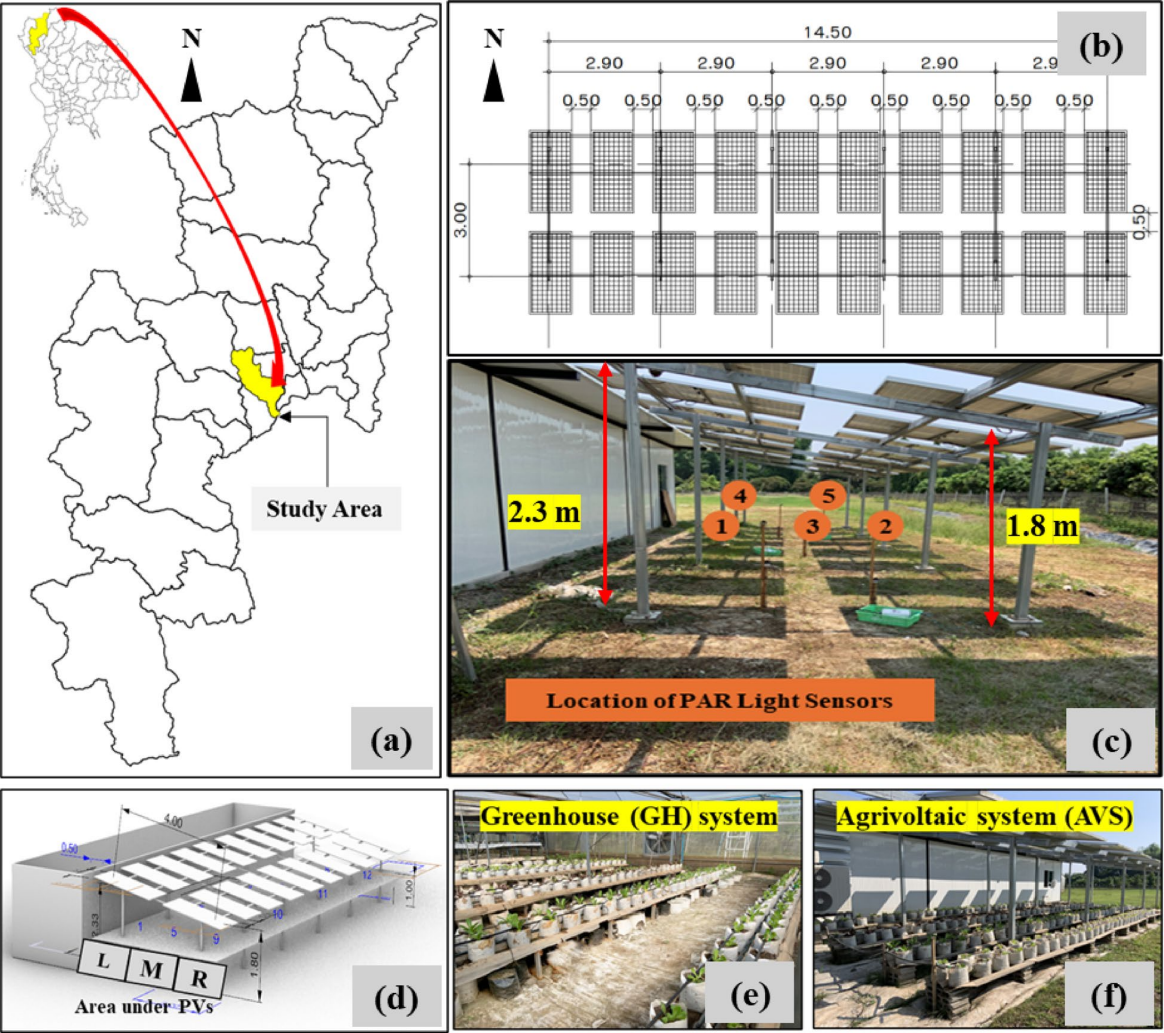
Location of the study area (map created using QGIS software, version 3.28.0; https://qgis.org/download/) (**a**), top-view design of PV system (**b**), overview of the ground mounted solar PV and location of installed PAR light sensors (**c**), the 3D design of the AVS created inRhinoceros 3D software representing three different sections of area underneath PVs (each section: 2.2 m in width and 16 m in length) (**d**), lettuce growing inside the GH system (**e**), and lettuce planting under the AVS (**f**). GH and AVS are two different planting systems as Greenhouse (GH) = lettuce planted inside the greenhouse conditions and Agrivoltaic system (AVS) = lettuce planting beneath a 10 kW PV system.

The study was conducted in Hang Dong District, Chiang Mai Province, Northern Thailand (Fig. 1a), with an average temperature of 27 °C and mean relative humidity of 71.5%. The ground-mounted photovoltaic (PV) system was installed. The lowest and highest points of PVs were 1.8 and 2.3 m, respectively and the inclination of the PVs was oriented at 15° in the direction of the south. The size of each PV was 1.1 m in width and 2.2 m in length. The total number of PVs was 20, with a system capacity of 10 kW and a spacing distance of 0.50 m apart from each other (Fig. 1b and c).

### Sunlight estimation under AVS and method validation

A PAR light sensor (WatchDog LightScout Quantum light sensor, Spectrum Technologies, Aurora, Illinois, USA) was used to quantify the sunlight between wavelengths of 400 and 700 nm. This measurement is known as photosynthetically photon flux density (PPFD, µmol m^−2^ s^−1^). One PAR sensor and one solar radiation sensor (W m^−2^; WatchDog LightScout Silicon Pyranometer) were installed at 1 m height above the ground outside the PVs, and the values were recorded every 60 min over September 2021 and August 2022. The recorded data of solar radiation and PPFD from the experiment was plotted to find correlations between these two data sets and the coefficient of determination (R^2^) was calculated. The R^2^ value of 0.94 indicated a very strong correlation between these two sets of data. The developed equation was then used to calculate the PPFD from the predicted solar radiation as follows:

1 $${\text{PPFD}}\,{\text{=}}\,{\text{1}}{\text{.7016 }}\left( {{\text{solar radiation}}} \right)\,{\text{+}}\,{\text{24}}{\text{.9}}$$

Sunlight availability underneath solar PV was estimated using the computer software Rhinoceros 3D (version 7; https://www.rhino3d.com/7/) with the Grasshopper Plugin and Ladybug Tools (version 0.0.67; https://www.food4rhino.com/en/app/ladybug-tools). The 3D dimensions of the PV system were drawn according to the existing design from the experimental site. A 3D design was comprised of (i) the space between solar PVs, (ii) the height of solar PVs, and (iii) the required height for model calculation, set at 1 m above ground. Input parameters for the software were the 3D design of the PV system and Typical Meteorological data (TMYx) from the Chiang Mai weather station, including variables such as sunshine hours, solar radiation, air temperature, and relative humidity. The weather data included average values from 2005 to 2015. The values from the Grasshopper Plugin algorithm calculation were delivered as solar radiation (kWh m^−2^) for the period of observation. The estimated values from the model were then converted to the PPFD using Eq. 1.

The sunlight estimation was conducted from 6 to 31 May 2022, covering 26 consecutive days. Hourly PPFD simulations were performed on a weekly basis to match plant growth characteristics. The first 21 days (6–26 May 2022) were used for model calibration, separated into three different weeks: (i) Week-I = 6–12 May 2022, (ii) Week-II = 13–19 May 2022, and (iii) Week-III = 20–26 May 2022. The remaining 5 days (Week-IV: 27–31 May 2022) were considered as the final week and these were used for model validation. From the predicted values, the data during the day (06:00–18:00) was used for model calibration and validation.

To verify the accuracy of the predicted values, five PAR light sensors were installed under the solar PVs at 1.0 m height above the soil surface and PPFD values were recorded every 60 min during the study period (Fig. 1c). Simulated and measured PPFD values were then compared.

### Monthly sunlight estimation

An estimation of PPFD values under the AVS was calculated for twelve months in a year. The total area under AVS was approximately 100 m^2^, and the area was divided into three different sections (L = left, M = middle, and R = right) as shown in Fig. 1d. Each section was 2.2 m in width and 16 m in length. Daily light integral (DLI, mol m^−2^ d^−1^) was calculated by multiplying the PPFD values with the light hours per day and by 0.0036 to convert from µmol m⁻² s⁻¹ to mol m⁻² d⁻¹, according to Eq. 2.

2 $${\text{DLI}}\,{\text{=}}\,{\text{PPFD} \times \text {light hours per day} \times 0}{\text{.0036}}$$

### Crop cultivation and data collection

Crop cultivation was tested underneath the solar PVs in order to investigate the potential of AVS to support both energy and food production. Experiments were conducted from November 2022 to February 2023. Lettuce (*Lactuca sativa* L.) was chosen due to its short growth cycle, high-value market demand, and suitability as a low DLI crop requirement. Three different lettuce varieties were selected for testing: ‘Butterhead’ (BH), ‘Green Oak’ (GO) and ‘Red Oak’ (RO). From Fig. 1e-f, two different planting systems were compared: (i) “Greenhouse (GH)” = lettuce planted inside the greenhouse conditions, setting for the control plot, and (ii) “Agrivoltaics (AVS)” = lettuce planting beneath a 10 kW PV system. The study area covered 1,600 m^2^ and the area of each planting system was approximately 100 m^2^. The spacing between plants was 25 cm and the total number of lettuce in 1 m^2^ was 16 plants.

Growing media was taken from the Faculty of Agriculture, Chiang Mai University, Chiang Mai, Thailand and the same growing media was applied for both planting systems. Four kg of growing media was put in each white growing bag (a diameter of 20 cm). Lettuce seedlings were transplanted into each growing bag at 14 days for both treatments, with a total lettuce of 120 plants per treatment (40 plants per variety per treatment, as seen in Fig. 1e-f). Drip irrigation was installed for each growing bag, and irrigation water was calculated according to crop evapotranspiration^26^. The total amount of irrigation water was 300 cm^3^ plant^−1^ d^−1^. Lettuces were planted in two cycles: the first cropping cycle (Crop I) was from 3 November 2022 to 30 November 2022, and the second cropping cycle (Crop II) was from 16 January 2023 to 12 February 2023.

Data collection was comprised of plant growth (plant height and canopy width) and yield (fresh and dry weight). The number of samples was four plants per lettuce variety at each planting system. Plant height (cm) and canopy width (cm) were measured every 7 days after transplanting (DAT) using a Vernier caliper. The fresh weight (FW, g plant^−1^) was collected every 7 DAT, and the samples were weighed directly after each harvesting time using a digital balance (0.01 g). The plant samples were dried at 65 °C for three days, and the dry weight (DW, g plant^−1^) was recorded.

For light availability, PAR light sensors were installed in three different locations: underneath the PVs, inside the GH treatment and under full sun. Then, the DLI values (mol m^−2^ d^−1^) were calculated using Eq. 2.

### Land equivalent ratio

To evaluate land use efficiency under AVS, the Land Equivalent Ratio (LER) was used to compare land utilization under different systems^27^. The LER is initially established to assess intercropping efficiency, but it has recently been employed to evaluate land use efficiency of the AVS in comparison to conventional agricultural practices or solar farms alone^28,29^. In this study, three different systems classified as GH, AVS and solar farm systems were compared. . The LER was calculated by summing the ratios of lettuce yield and energy income obtained from the AVS compared to crop cultivation or solar farm alone, according to Eq. 3. A value greater than 1 indicates more efficient land productivity.

3 $${\text{LER = }}\left( {{\text{F}}{{\text{W}}_{{\text{AVS}}}}{\text{/ F}}{{\text{W}}_{{\text{GH}}}}} \right){\text{ + }}\left( {{\text{Electricity incom}}{{\text{e}}_{{\text{AVS}}}}{\text{/ Electricity incom}}{{\text{e}}_{{\text{Solar farm}}}}} \right)$$

### Statistical analysis

The model accuracy was evaluated using the coefficient of determination (R^2^) and root mean square error (RMSE). Linear regressions were also conducted to develop the calibration and validation models. For experimental data, descriptive statistics, including means and standard deviations, were calculated for each lettuce variety within each planting system. At maturity (Week 4), comparisons between means of the two different planting systems were estimated using independent sample Welch’s t-test. Moreover, the Pearson correlation coefficient (r) and linear regression were used to analyze the relationships between cumulative DLI (mol m^−2^) and yield (fresh and dry weight, g plant^−1^). All the statistical analyses were performed using Microsoft Excel (Microsoft Office 365).

## Results

### Simulated sunlight under AVS and model validation

**Fig. 2:**
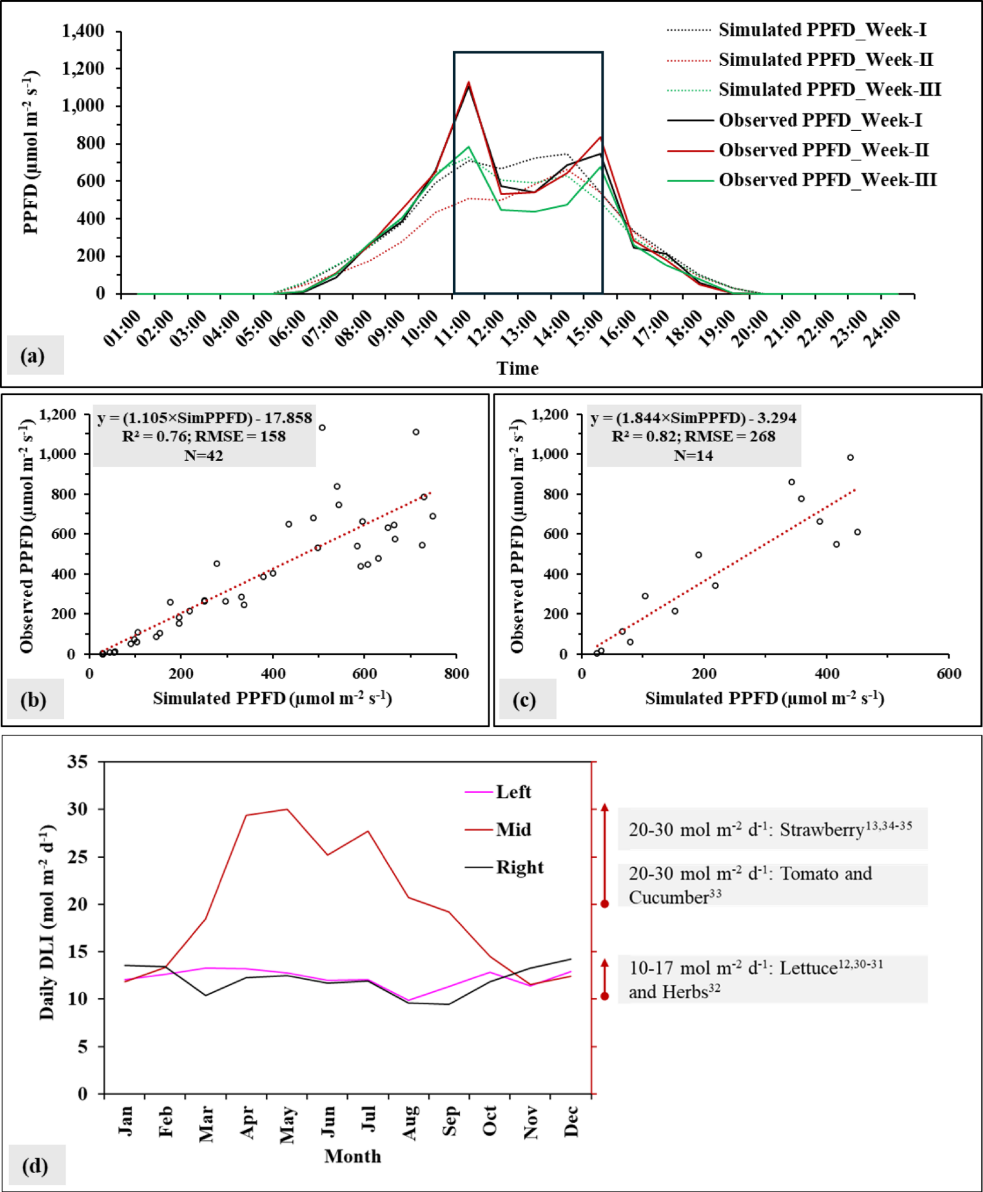
(**a**) Both simulated and observed hourly PPFD of three weeks, (**b**) model calibration of dataset between simulated and observed PPFD, (**c**) model validation of dataset between simulated and observed PPFD, and (**d**) calculated values of average daily DLI for one year, with recommended DLI for some crops^12,13,30–35^. R^2^ represents the coefficient of determination and RMSE indicates root mean square eror. N is the number of investigations.

Figure 2a shows the hourly PPFD, both simulated and observed values, over a three-week period. The simulated values corresponded with the observed data; however, higher values of PPFD were seen with the observed data in comparison to the simulated data. The highest peak of both simulated and observed PPFD occurred during the day between 10:30 am and 15:00 pm. Moreover, the results of the observed PPFD under the AVS fluctuated more throughout the day due to the shading effect of the PVs.

Model validation of light prediction under specific conditions showed that the relationship between simulated and observed PPFD displayed a moderate correlation between those two datasets, with R^2^ of model calibration 0.76 and model validation 0.82 (Fig. 2b-c). Higher RMSE was observed under both model calibration (158 µmol m^−2^ s^−1^) and validation (268 µmol m^−2^ s^−1^). However, correlations between the datasets demonstrated the capacity and accuracy of the model in predicting PPFD values. The developed equation from model validation was then used for converting the predicted solar radiation to PPFD (Fig. 2c).

Variations of the calculated annual profile of average daily DLI were predicted over a year (Fig. 2d). This showed the seasonal changes in sunlight availability underneath the solar panels of three different sections. The highest DLI was in the middle area compared to the left and right sections. However, the differences in DLI values during the dry season were minimal in January, February, November, and December. The average DLI value was 15.4 mol m^−2^ d^−1^ for all three sections, with the average values at each section of left = 12.6 mol m^−2^ d^−1^, middle = 20.6 mol m^−2^ d^−1^ and right = 12.9 mol m^−2^ d^−1^. The simulated DLI values were compared to the recommended thresholds for optimal growth of specific crops, such as lettuce, herbs, tomato, cucumber and strawberry. These results gave important information on an accurate quantification of available light energy and highlighted periods of excess or deficit relative to crop-specific light requirements. For example, lettuce and herbs require DLI between 10 and 17 mol m^−2^ d^−1^ and the simulated values of DLI were found to be sufficient to grow crops underneath the PVs.

### Variation of sunlight availability, crop growth and yield under different systems

**Fig. 3:**
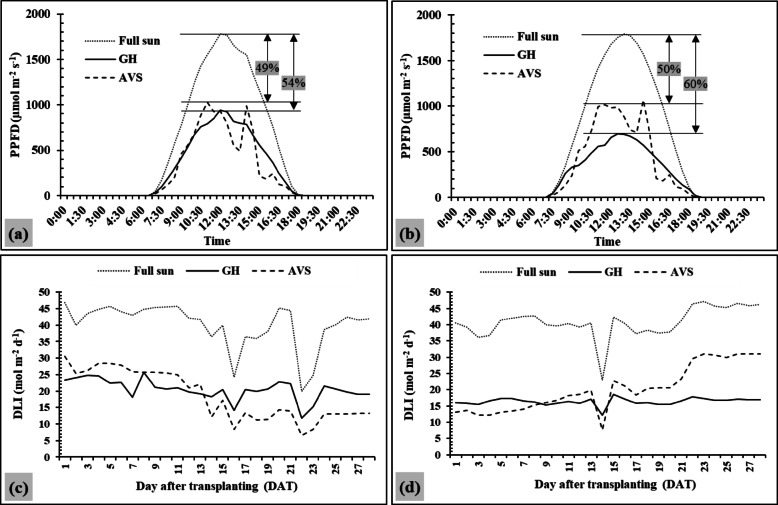
Average hourly PPFD for the first (**a**) and second (**b**) cropping cycles under different production systems. Average daily DLI under different systems for the first (**c**) and second (**d**) cropping cycles. GH and AVS are two different planting systems as Greenhouse (GH) = lettuce planted inside the greenhouse conditions and Agrivoltaic system (AVS) = lettuce planting beneath a 10 kW PV system.

For experimental results, the average sunlight availability (PPFD and DLI) under different planting systems showed similar trends under two cropping periods (Fig. 3). During the measurement period, the full sun setup measured the highest values of PPFD. The peak PPFD for both cropping periods was found during the day between 10:30 and 15:00 h. Sunlight reduction was observed in the GH (54% in Crop-I and 60% in Crop-II) and the AVS (49% in Crop-I and 50% in Crop-II) in comparison to the full sun. Variations of sunlight under the AVS showed fluctuating trends during the day due to shading effects.

Figure 3c-d shows the average daily DLI values over two cropping periods. The results indicate that the DLI values were highest in the full sun (40 mol m^−2^ d^−1^ in Crop I and 41 mol m^−2^ d^−1^ in Crop II) in comparison to the GH (20 mol m^−2^ d^−1^ in Crop I and 16 mol m^−2^ d^−1^ in Crop II) and AVS (19 mol m^−2^ d^−1^ in Crop I and 20 mol m^−2^ d^−1^ in Crop II). The AVS tended to have similar DLI values compared to the GH system during the study periods under both cropping systems.

**Fig. 4:**
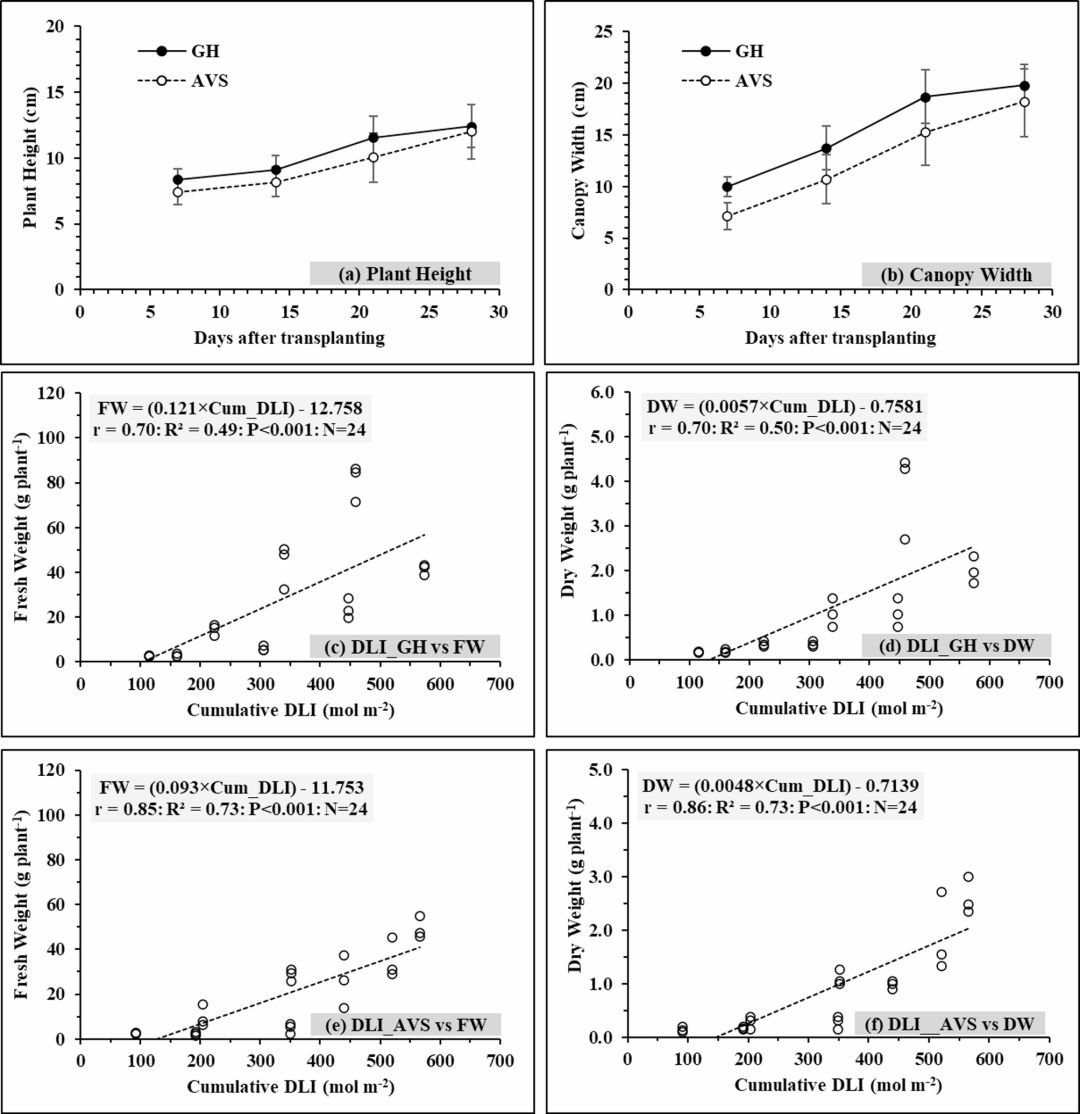
Plant height (**a**) and canopy width (**b**) over two crop cycles under different planting systems. The means and standard deviations are represented by data points and error bars. Relationships of (**c**) cumulative DLI and FW (**d**) cumulative DLI and DW in the GH planting system for all varieties, (**e**) cumulative DLI and FW and (**f**) cumulative DLI and DW under AVS planting system for all varieties. . GH and AVS are two different planting systems as Greenhouse (GH) = lettuce planted inside the greenhouse conditions and Agrivoltaic sysytem (AVS) = lettuce planting beneath a 10 kW PV system. FW is fresh weight and DW indicates dry weight. When r and R^2^ represent the Pearson correlation coefficient and coefficient of Ddetermination, respectively. N is the number of investigations.

Average values of plant height and canopy width were compared between the GH system and AVS over two crop cycles (Fig. 4a and b). At week 4 (28 DAT), 

**Table 1:** Mean and standard deviation (SD) values of plant height, canopy width and biomass production of lettuces at 28 DAT.

Parameters	Types	Crop cycles	Planting systems		t-value	*P*-value
			GH	AVS		
*Plant Growth (Mean ± SD)*						
Plant Height (cm) (*N* = 4)	BH	I	10.9 ± 0.5	10.4 ± 0.5	−1.477	0.190^ns^
		II	14.0 ± 0.7	9.4 ± 0.6	−9.773	0.000*
	GO	I	12.1 ± 0.6	14.8 ± 0.9	4.905	0.004*
		II	14.8 ± 0.6	12.4 ± 0.9	−4.416	0.009*
	RO	I	10.8 ± 0.3	13.5 ± 1.7	3.132	0.052*
		II	12.0 ± 0.4	11.5 ± 1.3	−0.739	0.501^ns^
	**Mean**		**12.4 ± 1.6**	**12.0 ± 2.1**	**0.816**	**0.419** ^**ns**^
Canopy Width (cm) (*N* = 4)	BH	I	18.0 ± 0.8	11.9 ± 1.5	−7.230	0.001*
		II	21.6 ± 2.3	17.6 ± 2.5	−2.384	0.055^ns^
	GO	I	19.3 ± 1.0	21.0 ± 1.3	2.050	0.086^ns^
		II	20.8 ± 0.7	21.1 ± 0.7	0.514	0.626^ns^
	RO	I	19.8 ± 0.6	19.8 ± 1.2	0.000	1.000^ns^
		II	19.3 ± 1.0	18.5 ± 2.1	−0.700	0.523^ns^
	**Mean**		**19.8 ± 1.6**	**18.3 ± 3.5**	**1.912**	**0.065** ^**ns**^
*Yield (Mean ± SD)*						
FW (g plant^−1^) (*N* = 4)	BH	I	43.2 ± 3.4	31.0 ± 6.9	−3.191	0.033*
		II	86.0 ± 9.2	45.6 ± 9.5	−6.117	0.001*
	GO	I	38.7 ± 5.6	45.3 ± 4.3	1.882	0.109^ns^
		II	84.7 ± 9.9	55.0 ± 9.4	−4.342	0.005*
	RO	I	42.5 ± 4.5	28.9 ± 4.1	−4.475	0.004*
		II	71.4 ± 15.4	47.1 ± 2.7	−3.104	0.053^ns^
	**Mean**		**61.1 ± 22.1**	**42.2 ± 11.1**	**3.742**	0.001*
DW (g plant^−1^) (*N* = 4)	BH	I	2.0 ± 0.1	1.5 ± 0.3	−3.249	0.048*
		II	4.4 ± 0.3	2.5 ± 0.6	−5.895	0.002*
	GO	I	2.3 ± 0.2	2.7 ± 0.2	2.429	0.051^ns^
		II	4.3 ± 0.4	3.0 ± 0.6	−3.927	0.011*
	RO	I	1.7 ± 0.4	1.3 ± 0.2	−1.732	0.144^ns^
		II	2.7 ± 1.0	2.4 ± 0.5	−0.672	0.538^ns^
	**Mean**		**2.9 ± 1.2**	**2.2 ± 0.7**	**2.396**	0.022*

GH and AVS are the different planting systems of lettuce growing in the greenhouse (GH) and under PVs (AVS).

BH, GO and RO are ‘Butter Head’, ’Green Oak’ and ‘Red Oak’, respectively.

FW and DW indicate fresh and dry weight, respectively.

N is number of investigation samples.

ns and * are non-significant and significant differences of means at *P* < 0.05.

Table 1 shows the mean values of plant growth (plant height and canopy width) and yield (FW and DW). The differences in plant height and canopy width were not significant between the two different planting systems. However, the GH system tended to have higher plant height (12.4 cm) and canopy width (19.8 cm) in comparison to the AVS (12.0 and 18.3 cm). In addition, a significant difference was found in FW between the two systems. The AVS had a lower average FW (42.2 g plant^−1^), compared to the GH system (61.1 g plant^−1^). The average value of DW in the AVS (2.2 g plant^−1^) was significantly lower than in the GH system (2.9 g plant^−1^).

### Relationships between yields and cumulative DLI

Pearson correlation coefficients (r) and coefficients of determination (R^2^) between DLI and yields (FW and DW) were calculated (Fig. 4c-f). Significant positive correlations were observed between cumulative DLI and yields (FW and DW) under both planting systems. The highest correlation *(r* = 0.86) was found between DLI and DW under the AVS, and the lowest correlation was found under the GH system between DLI and yields (FW and DW) at *r* = 0.70. The AVS had greater correlations between DLI and yields (*r* = 0.85 in DLI vs. FW and *r* = 0.86 in DLI vs. DW) in comparison to the GH system (*r* = 0.70 in DLI vs. FW and *r* = 0.70 in DLI vs. DW). 

In addition, the R^2^ values from the regressions were found to be significant in both planting systems. In the GH system, relationships between cumulative DLI and yields were moderate, with R^2^ = 0.49 of DLI vs. FW and R^2^ = 0.50 of DLI vs. DW.  On the other hand, the AVS showed stronger relationships compared to the GH system (R^2^ of 0.73 for DLI vs. FW and 0.73 for DLI vs. DW). This demonstrated that the developed equation can predict FW and DW of lettuces, with an accuracy of approximately 49-50% under the GH system and about 73% under the AVS.

### Annual lettuce yield prediction under AVS

**Fig. 5:**
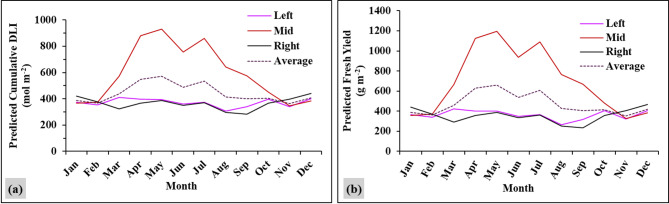
Predicted monthly cumulative DLI (**a**) and monthly lettuce yield prediction (**b**) under the AVS. Left, mid and right indicate left, middle and right sections of the area underneath PVs (AVS). DLI refers to daily light integral. The monthly cumulative DLI was calculated as the predicted daily DLI values (Fig. 2d) multiplied by the number of days in each respective month. The monthly lettuce yield prediction was estimated using the equation presented in Fig. 4e multiplied by 16 plants m⁻² .

From data in Figs. 2c and 4e, equations were used to estimate both monthly cumulative DLI [PPFD=(1.844×Sim PPFD) – 3.249] and yields [FW=(0.093×Cum DLI) – 11.753]. Figure 5 shows the seasonal variations in predicted lettuce yield under the AVS throughout the year. The average highest and lowest predicted yield were observed in May (776 g m⁻²) and November (325 g m⁻²), respectively. This variation is associated with the changes in cumulative DLI, which is also highest in May (648 mol m⁻²) and lowest in November (344 mol m⁻²). The predicted yield fluctuates across the months, with higher values during periods of increased light intensity and lower values during months with reduced sunlight (northern winter).

Among the different sections of the AVS, the middle area consistently produces the highest predicted yield over the year in comparison to the other sections (average of 747 g m^−2^). The left and right sections exhibited similar trends, with average predicted yields of 381 g m⁻² and 397 g m⁻², respectively. The middle section had the highest predicted yield in almost all months, producing 60–70% more in the months with high cumulative DLI than the side sections.

### Land equivalent ratio (LER) under different systems

The GH system had the highest total income (296,212 USD ha^−1^ year^−1^) when compared to the AVS (251,123 USD ha^−1^ year^−1^) and solar farm (108,588 USD ha^−1^ year^−1^), according to Table 2. However, the LER value under the AVS was higher than the other systems, with a value of 1.12. This indicated that a higher performance of the land by about 12% was observed in comparison to single-use systems.

**Table 2:** Land equivalent ratio under different systems.

Parameters	Systems		
	GH	AVS	Solar Farm
1. Lettuce yield (g plant^−1^)	61.1^a^	42.2^a^	0
2. Lettuce yield (kg ha^−1^ year^−1^)	97,760^a−d^	67,520^a−d^	0
3. Lettuce income (USD ha^−1^ year^−1^)^c,d^	296,213	204,586	0
4. Energy income (USD ha^−1^ year^−1^)	0	46,538^c, e^	108,588^c, f^
5. Total income (USD ha^−1^ year^−1^)	296,212	251,124	108,588
6. **Land Equivalent Ratio (LER)**	**1.00**	**1.12**	**1.00**

^a^ Data taken from experiment under the GH and AVS systems, with values of 61.1 and 42.2 g plant^−1^, respectively.

^b^ The spacing between plants is 25 cm and the total numbers of lettuce in 1 m^2^ is 16 plants (160,000 plants ha^−1^), with ten cropping cycles per year.

^c^ 1 USD = 33 Thai Baht.

^d^ Average lettuce price = 3.03 USD kg^−1^ based on local market prices in Thailand^36^.

^e^ AVS: Energy revenue is calculated with a system efficient loss of 15% and electricity price of 0.10 USD^37^ kWh^−1^, with five sunlight hours per day and PVs were covered 30% of the areas.

^f^ Solar Farm: Energy revenue is calculated with a system efficient loss of 15% and electricity price of 0.10 USD^37^ kWh^−1^, with five sunlight hours per day and PVs were covered 60% of the areas.

## Discussion

The findings of this study highlighted the potentiality of using models to predict the light quality under specific conditions and the efficiency of implementing AVS to grow test crops (lettuce). The Rhinoceros 3D software with the Grasshopper Plugin and Ladybug Tools revealed a possibility of the software to simulate sunlight availability under the AVS system. The regressions between the simulated and observed PPFD values showed strong correlations (R² = 0.76 for calibration and R² = 0.82 for validation), indicating the robustness of the prediction model. These results were correlated to another study  ^24^, which employed similar tools to estimate the light distribution on vertical shelves, and the results showed a strong capability to predict PPFD with R^2^ = 0.87. However, errors also increase in the high-light range (RMSE = 158 µmol m^-2^ s^-1^ for model calibration and RMSE = 268 µmol m^-2^ s^-1^ for model validation). Accurate light simulation is useful for optimizing crop production and supporting effective crop planning and management. This is particularly important for the AVS, where the shading effect of PV panels can create significant changes in light patterns. Moreover, an assessment of sunlight variations under AVS conditions can help to adjust crop types, planting density, and positions throughout the growing season.

The simulated results displayed the annual DLI values under the different sections of the AVS (Fig. 2). The middle section of the system received more light than the other parts. This temporal heterogeneity of sunlight underlines the importance of crop scheduling or crop selection to optimize the light availability for crops during the year. Variations in sunlight under the solar PVs can be influenced by various factors such as the angle and spacing of the PV panels, the time of year, and the geographical location.

From Fig. 4e-f, strong correlations between the cumulative DLI and crop yield (both FW and DW) were observed under the AVS (R^2^ = 0.73). This suggests that light conditions (fluctuating trends) under the AVS have the potential to accurately predict yield outcomes. On the other hand, the relatively low R^2^ values were detected under the GHsystem, showing a weak relationship between cumulative DLI and yield. These results demonstrate that in controlled environments with constant light, additional environmental factors, such as temperature, humidity, or CO₂ concentration, may have a more important impact on crop productivity. Subsequently, using the values of cumulative DLI alone might not be sufficient to predict yields under such conditions. This finding was related to the results of another study ^33^, which reported that even when the total DLI remained constant, the temporal distribution of light, for example, photoperiod or continuous lighting, had a substantial effect on physiological responses and yields. Therefore, cumulative DLI can be used for lettuce yield prediction under the AVS, but additional environmental factors should be considered to improve yield prediction accuracy under the GH system.

The results of monthly lettuce yield prediction based on cumulative DLI can give additional benefits for production planning and economic forecasting under the AVS (Figs. 4e and 5). The seasonal variations of predicted yield show that months with abundant light, like May, were associated with higher yields. During periods of limited light, such as in November, it was also restricted to lower yields. The middle section consistently produced higher yields than the left and right sections. These trends can be used for crop planting schedules and linked to the market demand and supply during the year. Therefore, integration of cumulative DLI-based yield predictions supports better management decisions on crop placement and time, especially in Thailand with strong seasonal changes in sunlight. The simulation results can help farmers to choose suitable crops for each season, decide the best planting time, and use the most productive sections efficiently. This supports planning of resources and reduces economic risks during low-light periods.

From the experimental results, growth parameters (plant height and canopy width) of lettuces showed significant differences between the AVS and GH systems during the measurement periods (Fig. 4a and b). This may be due to similarly reduced light intensities as both systems received less sunlight (almost 49–60%) compared to the open-field condition. Similar results were seen in the another study ^38,^ which reported that lettuce grown under 50% shade nets showed increased leaf areas and longer stems in comparison to open-field cultivation. Such morphological responses are characterized by shade-avoidance syndrome^14,21,39^. Average FW (GH = 61.1 g plant^−1^ and AVS = 42.2 g plant^−1^) under both systems was lower than the other studies conducted with lettuce under greenhouse conditions in Thailand^39–41^. These might be due to several factors, such as different varieties, growing media, and cultivation methods. The results implied that light distribution in the GH had less direct effect on yield compared to the AVS. In the GH, other factors such as temperature and humidity may play a more significant role, reducing the direct influence of DLI on yield. In addition, soil conditions and pathogen pressure may also contribute to yield variations and should be considered together with light and microclimate factors.

Moreover, the FW under the AVS was significantly lower than the GH system in this study. Even though the light amounts under both systems were slightly different (DLI = 18 mol m^−2^ d^−1^ under the GH system and DLI = 19 mol m^−2^ d^−1^ under the AVS), these variations could be attributed to differences in light distribution and plant light interception during the day. Minor variations in light measurements between the GH and AVS systems were found between the first and second crop cycles, possibly due to the 46-day interval between harvesting cycles. This caused a change in the position of the sun in the sky at the same hour of the day, and the altered position of the sun affected the shadow in the area beneath the AVS. Moreover, sunlight characteristics under the AVS fluctuated throughout the day due to intermittent shading from the PV panels (Figs. 2 and 3). This was correlated to other studies ^21,42^, which pointed out that light distribution under PVs was not uniform during the measurement. The rapid changes of sunlight between intense light and deep shade can induce physiological light stress for lettuce, as plants cannot adjust their photosynthetic process immediately. These fluctuating light conditions, in which PPFD values may exceed the photosynthetic saturation threshold for lettuce for some time (~ 500 µmol m⁻² s⁻¹) ^16)^ and then may fall below optimal ranges. This can affect overall photosynthetic efficiency; for example, plants will require more time for the photosynthetic process to adapt to the rapid changes in light intensity^43,44^. On the other hand, the GH system maintained more stable light conditions within the optimal range for lettuce growth during the day. These fluctuating light intensities may have limited photosynthetic efficiency and contributed to the lower yields observed under the AVS.

According to Table 2, the LER value was lower than the other studies^45,46^. However, the LER under the AVS (1.12) was higher than the other systems, indicating the higher performance of the land by about 12% compared to single-use systems. These findings emphasize the potential of the AVS as the dual land use system for both food and energy production. Other factors like investment & operational costs and energy consumption represent important aspects of system performance. Crop production under the GH system usually requires higher operational energy for cooling, ventilation, and irrigation, meanwhile, the AVS can generate renewable electricity and reduce external energy demand. Moreover, carbon benefits of AVS result from renewable power generation. This increases the potential of the AVS to lower GHG emissions from using fossil fuel.

For research limitations and future study, the results help to understand light variation and crop production under the AVS, however, this study is subject to certain limitations as follows:

- The experiment was conducted during the cool-dry season in Thailand, and it did not reflect year-round crop production.
- Crop selection was only focused on lettuce, and this did not represent the other crops under different light requirements.
- Microclimate data, such as temperature and wind, was not fully investigated.

Therefore, future research is needed in order to address these limitations, especially in Thailand, following these directions:

- Long-term prediction: Extending the study to a full annual cycle would capture seasonal variability, which can yield a more comprehensive dataset. Broader calibration and validation should improve robustness and accuracy of the model. This is expected to reduce error metrics such as lower RMSE.
- System design and crop selection: The study should explore an optimal spacing and height of the PV installation, prediction of the light distribution under the PVs, and selection of shade-tolerant crops.
- Crop diversity: Increasing crop varieties planted under AVS conditions will help to understand the species-specific responses to shaded environments.
- Plant physiology and soil property study: The research should be conducted on plant response to shaded conditions, soil-and-plant relationships, and long-term impacts on soil properties (such as soil moisture and microbial activity).
- Economic analysis: In-depth evaluation of investment cost, maintenance and operational costs, together with potential returns, should be investigated.

## Conclusion

This study demonstrated the ability of the Rhinoceros 3D software with the Grasshopper Plugin and Ladybug Tools to accurately predict sunlight variations under the AVS conditions. The results showed that the model could provide valuable information on the spatial and temporal variations of sunlight under the PV both daily and monthly throughout the year. This information can be used to optimize crop management. Experimental results showed that light intensity under solar PVs (AVS) was enough for lettuce plantation, even though lower yield was observed in comparison to the GH system. The land use efficiency was 12% (LER = 1.12) better than the other systems. These showed the potential of the AVS as a dual land use system for both food and energy production. However, future studies should be investigated including the topics of system design and crop selection, crop diversity, plant physiology and soil properties, and economic analysis, in order to provide information and contribute to the development of the AVS in local agricultural frameworks.

## Data Availability

The data that support the findings of this study are available from the corresponding author upon reasonable request.
